# Medical and allied health service use during acute and chronic post-injury periods in whiplash injured individuals

**DOI:** 10.1186/s12913-020-05146-0

**Published:** 2020-03-30

**Authors:** Carrie Ritchie, Ashley Smith, Michele Sterling

**Affiliations:** 1 Recover Injury Research Centre, The University of Queensland, Brisbane, Queensland Australia; 2 School of Allied Health Sciences, Griffith University, Gold Coast, Australia; 3 Department of Clinical Neurosciences, Cumming School of Medicine, University of Calgary, Calgary, Canada; 4 NHMRC Centre of Research Excellence in Road Traffic Injury Recovery, Brisbane, Australia

**Keywords:** Whiplash injuries, Neck pain, Radiology, Physical therapist, Health services, Acute pain, Chronic pain, Medicine, General practitioners

## Abstract

**Background:**

Individuals with whiplash associated disorder (WAD) frequently experience neck pain in addition to other physical, psychological and social symptoms. Consequently, treatment is sought from a variety of health professionals. The limited data available about health services use in this population are conflicting. This study aimed to characterise health service use in individuals with WAD from a motor vehicle crash.

**Methods:**

Medical (general practitioner (GP), medical specialist, emergency services (ED), radiology – x-ray, computed tomography, magnetic resonance imaging, ultrasound) and allied health service (physiotherapy, chiropractor, psychologist, osteopath, occupational therapy) use during acute (< 12 weeks) and chronic (12 weeks to 2 years) post-injury periods were analysed in adults claiming compensation for WAD in the no-fault jurisdiction of Victoria, Australia (*n* = 37,315).

**Results:**

Most WAD claimants had an acute post-injury health service payment (95%, *n* = 35,348), and approximately one-third (29%, *n* = 10,871) had a chronic post-injury health service payment. During an acute post-injury period, the most frequently compensated services were for: ED (82% of acute claimants), radiology (56%), and medical specialist (38%). Whereas, physiotherapy (64.4% of chronic claimants), GP (48.1%), and radiology (34.6%) were the most frequently paid services during the chronic period. Females received significantly more payments from physiotherapists (F = 23.4%, M = 18%, z = − 11.3, *p* < .001, r = 0.13), chiropractors (F = 7.4%, M = 5.6%, z = − 6.3, *p* < .001, r = 0.13), and psychologists (F = 4.2%, M = 2.8%, z = − 6.7, *p* < .001, r = 0.18); whereas, males received significantly more medical services payments from medical specialists (F = 41.8%, M = 43.8%, z = − 3.7, *p* < .001, r = 0.03), ED (F = 74.0%, M = 76.3%, z = − 4.9, *p* < .001, r = 0.03) and radiology (F = 58.3%, M = 60.1%, z = − 3.4, *p* < .001, r = 0.02).

**Conclusions:**

Individuals with WAD claimed for a range of health services. Radiology imaging use during the acute post-injury period, and physiotherapy and chiropractor service use during the chronic post-injury period appeared concordant with current WAD management guidelines. Conversely, low physiotherapy and chiropractic use during an acute post-injury period, and high radiology and medical specialists use during the chronic post-injury period appeared discordant with current guidelines. Strategies are needed to help inform medical health professionals of the current guidelines to promote early access to health professionals likely to provide an active approach to treatment, and to address unnecessary referral to radiology and medical specialists in individuals with on-going WAD.

## Background

Whiplash-associated disorder (WAD) is the term used to describe an array of symptoms incurred from an acceleration/deceleration injury to the neck, usually following a motor vehicle crash (MVC). WAD is the most common and costly of all survivable MVC injuries [1]. Approximately 50% of individuals with WAD fully recovery while the remaining 50% continue to experience some level of on-going pain or disability [2, 3]. Although neck pain is the most frequent symptom, it is common for individuals with WAD to experience other physical, psychological, and social symptoms [4, 5]. Consequently, injured individuals may seek treatment from a variety of health professionals for pain as well as other symptoms.

Clinical guidelines for WAD management focus on treatment during the acute post-injury period since most recovery, if it occurs, takes place in the initial 3 months post-injury with little improvement after this time point [2, 6]. Acute clinical WAD management guidelines recommend that health service providers provide: reassurance; advice to stay active and return to usual activities as soon as possible; and, if needed, simple analgesic and non-steroidal anti-inflammatory medicines. In addition, these guidelines recommend against passive treatments such as immobilisation with a cervical collar, surgery or pharmacological injections [7–10]. The limited clinical guidelines for individuals with on-going WAD (e.g. > 3 months post-injury) also recommend exercise and activity management, and if psychological symptoms are present, the possible addition of behavioural and psychological strategies [9, 10]. To our knowledge, there are no data available that delineate acute from chronic health service use by individuals with WAD.

Timing and volume of health service use after an acute whiplash injury appear to affect health outcomes, healthcare utilisaton and costs [11–13]. Individuals with WAD who waited more than 28 days after their injury to consult a physiotherapist reported greater healthcare use compared with those who consulted a physiotherapist within 28 days of their injury [14]. Additionally, individuals with neck pain who waited > 90 days to consult a physiotherapist accessed more guideline discordant health services (e.g., spinal injections) compared with individuals who consulted a physiotherapist within 14 days of a new eipsode of neck pain [11]. On the other hand, overtreatment by healthcare providers in the first 3 months following a whiplash injury has been shown to slow recovery [12, 13].

The limited data available about the types of health services used by this population are conflicting. Survey results showed that over one-half of emergency and primary health medical practitioners indicated that they would refer patients with WAD to physiotherapists [15, 16], orthopaedic specialists [16], and psychologists [16]. However, audits of general practitioner (GP) management of individuals with WAD in Australia [17] and insurance claimant data in Canada [12] found that less than one-quarter of individuals were referred to physiotherapists and medical specialists. There appears to be no data available about referral of individuals with WAD to psychologists. Although optimal management of WAD is not yet known, to ensure compliance with current WAD management guidelines, a better understanding of current health service utilization during acute and chronic post-injury periods is needed. This study aimed to characterise health service use during acute and chronic post-injury periods in individuals with WAD from a MVC. In addition, we aimed to identify factors predictive of: a) likely recovery (health care service utilization only during acute phase); and b) high health care service utilization (highest quartile of overall health service use).

## Methods

Participants were not recruited to this study directly. De-identified data from the Compensation Research Database (CRD) were provided by the Institute for Safety, Compensation and Recovery Research in Victoria, Australia, an initiative funded by the Transport Accident Commission (TAC) (https://www.tac.vic.gov.au/). The TAC is a government organisation within the state of Victoria, the second most populous state in Australia. The TAC pays for the treatment and benefits of all individuals (i.e. vehicle occupants, cyclists and pedestrians) involved in an accident caused by a motorised vehicle registered in the state of Victoria. The injured person (or representative) generally has 12 months from date of injury to initiate a claim for compensation. The existent no-fault compensation system within Victoria means that injury-related medical and rehabilitation costs are compensated whether or not the claimant is considered to be at fault for the accident. However, most medical and allied health treatment costs incur a medical excess. A claimant must pay the medical excess before being eligible for reimbursement from the TAC. The medical excess amount varied during the study period from AU$450 (in 2000) to AU$599 (in 2013). For this specific study, data were analysed for medical and allied health service claims only. Pharmaceutical claim payments have been analysed previously [18].

### Participants

Data were provided for all claimants with a transport related injury date between 1 January 2000 and 31 December 2013 who met the following inclusion criteria: (1) > 18 years; (2) most serious injury coded by the TAC was a whiplash injury (i.e. cervical spine strain with or without minor physical injuries to other parts of the body, e.g. contusions/laceration), (3) were not admitted to hospital as a result of their transport related injury; and (4) received a claim payment for at least one health service (i.e. medical or allied health service).

For each individual, data were extracted for all medical (e.g. emergency services, general practitioners, medical specialists, radiology), and allied health service (e.g. physiotherapy, chiropractic, osteopathy, psychology, occupational therapy) claims paid during the life of their claim or up to a maximum of 2 years post injury. Human research ethics approval was granted from The University of Queensland (#2017001361/2016/112).

### Data

Demographic data included: sex; age at the time of MVC; area of residence (major cities, inner regional, outer regional, remote or very remote) [19]; year of MVC; and whether or not the claimant had lodged a common law claim. A common law claim is a court action commenced within 6 years of the date of injury that requires the claimant to have an injury deemed to be ‘serious’ and to be able to show that another person was at fault. An acute claim payment was defined as a health service provided within 12 weeks of the MVC, and a chronic claim payment was defined as a health service provided between 12 weeks and 2 years post-MVC.

Paid health service claims were grouped into one of nine categories that comprised allied health services from: (1) physiotherapists, (2) chiropractors, (3) osteopaths, (4) psychologists, and (5) occupational therapists (OT); and medical services from: (6) general practitioners (GP), (7) medical specialists, (8) emergency services, and (9) radiology services. Radiology services were further specified as: plain radiograph (x-ray), computed tomography scans (CT), magnetic resonance imaging scans (MRI), and ultrasound.

### Analyses

Specific health service and imaging use were described by the demographic factors: age, sex, area of residence, year of MVC, and lodgement of common-law claim for both prevalence (e.g. the number of health service group claimants divided by total WAD claimants) and as an aggregate (e.g. the number of specific health service sessions among users). Health service use was also described for post-injury claim phases (e.g., acute or chronic post-injury phase). All variables were non-normally distributed, hence non-parametric statistics were used to compare demographic data between groups. Logistic regression was used to determine predictive factors associated with two health service use profiles: 1. likely recovery, e.g. only acute post-injury health service claims; and 2. high health care service utilization, e.g. the highest quartile of overall health service claims. The demographic factors outlined above were the possible predictive factors.

## Results

### Health service use

Between 1 January, 2000 and 31 December, 2013, 37,315 individuals were paid a compensation claim for a health service following a whiplash injury through the TAC in Victoria, Australia. Approximately two-thirds of claimants were female, and the majority lived in a major city (Table 1). One-quarter of claimants were aged 18–24 years and claims incidence decreased with increasing age. Only 4% of claimants lodged a common law claim (Table 1). Three in four claimants received a compensation payment for emergency services and three in five for a radiology claim (Table 1). Of the health professional service categories, medical specialists were seen by the largest number of claimants followed by GPs (Table 1). Only one in five claimants received compensation payment for physiotherapy services and less than one in ten for chiropractic service. There were no differences in the proportions of males and females who claimed for health services from GPs. However, compared with males, females were significantly more likely to receive a compensation payment for all allied health services (e.g. physiotherapists, chiropractors, osteopaths, psychologists, and OT); whereas, males were more likely receive a compensation payment for medical services from medical specialists, emergency services and radiology (Table 1). Comparison of health service use by age group revealed that: claimants in the youngest age category (18–24 years) were less likely to receive payment for all services except emergency services; claimants aged 45–64 years were more likely than younger and older groups to receive payment for physiotherapy, GP and medical specialist services; and claimants in the oldest age group (> 65 years) were more likely than younger age groups to receive payment for radiology (Table 1). Health service use by area of residence showed that claimants from major cities were significantly more likely to receive payment for medical specialist services compared with regional and remote areas, whereas claimants from regional and remote areas were more likely to receive payment for GP services compared with claimants from major cities (Table 1). In addition, claimants from major cities were almost twice as likely to receive payment for physiotherapy and psychology services compared with claimants from regional areas. Individuals who lodged a common law claim were significantly more likely to receive a compensation claim for all health services except emergency services compared with claimants who did not lodge a common law claim (Table 1).

**Table 1:** Number (percentage) of claimants receiving payments for each specific health service categories by descriptive category

	Total TAC claimants N (column %)	Allied Health Services					Medical Services			
		Physiotherapy	Chiropractic	Osteopathy	Psychologist	OT	GP	Medical specialist	Emergency services	Radiology
**Total (row percentage)**	37,315	8087 (21.7%)	2521 (6.8%)	1233 (3.3%)	1386 (3.7%)	745 (2.0%)	15,443 (41.4%)	15,854 (42.5%)	27,919 (74.8%)	21,975 (58.9%)
**Sex**										
Male	13,122 (35.2%)	2416 (18.4%)	741 (5.6%)	273 (2.1%)	371 (2.8%)	188 (1.4%)	5444 (41.5%)	5744 (43.8%)	10,014 (76.3%)	7881 (60.1%)
Female	24,182 (64.8%)	5669 (23.4%)	1778 (7.4%)	959 (4.0%)	1015 (4.2%)	557 (2.3%)	9996 (41.3%)	10,107 (41.8%)	17,900 (74.0%)	14,088 (58.3%)
Missing N=	11	2	2	1	0	0	3	3	5	6
z statistic (Mann-Whitney U), p, r^a^		z = −11.3, *p* < .001, r = 0.13	z = −6.3, *p* < .001, r = 0.13	z = −9.7, *p* < .001, r = 0.28	z = −6.7, *p* < .001, r = 0.18	z = −5.7, *p* < .001, r = 0.21	z = − 0.30, *p* < .777, r = 0.00	z = − 3.7, *p* < .001, r = 0.03	z = − 4.9, *p* < .001, r = 0.03	z = − 3.4, *p* < .001, r = 0.02
**Age**										
18–24	8981 (24.1%)	1022 (11.4%)	424 (4.7%)	168 (1.9%)	147 (1.6%)	57 (0.6%)	3326 (37.0%)	3692 (41.1%)	7488 (83.4%)	5411 (60.2%)
25–34	8632 (23.1%)	1791 (20.7%)	652 (7.6%)	347 (4.0%)	268 (3.1%)	136 (1.6%)	3259 (37.8%)	3612 (41.8%)	6480 (75.1%)	4824 (55.9%)
35–44	7269 (19.5%)	1910 (26.3%)	624 (8.9%)	313 (4.3%)	390 (5.4%)	221 (3.0%)	3212 (44.2%)	3064 (42.2%)	5118 (70.4%)	4206 (57.9%)
45–54	5941 (15.9%)	1688 (28.4%)	449 (7.6%)	222 (3.7%)	322 (5.4%)	163 (2.7%)	2707 (45.6%)	2649 (44.6%)	4177 (70.3%)	3576 (60.2%)
55–64	3676 (9.9%)	1058 (28.8%)	252 (6.9%)	114 (3.1%)	179 (4.9%)	99 (2.7%)	1714 (46.6%)	1611 (43.8%)	2528 (68.8%)	2233 (60.7%)
> 65	2816 (7.5%)	618 (21.9%)	120 (4.3%)	69 (2.5%)	80 (2.8%)	69 (2.5%)	1225 (43.5%)	1226 (43.5%)	2128 (75.6%)	1725 (61.3%)
Chi-squared (χ^2^) (Kruskal-Wallis H), p, ε^2a^		χ^2^ = 924.1, *p* < .001, ε^2^ = 0.11	χ^2^ = 140.3, *p* < .001, ε^2^ = 0.06	χ^2^ = 104.8, *p* < .001, ε^2^ = 0.09	χ^2^ = 240.8, *p* < .001, ε^2^ = 0.17	χ^2^ = 162.4, *p* < .001, ε^2^ = 0.22	χ^2^ = 230.1, *p* < .001, ε^2^ = 0.01	χ^2^ = 23.5, *p* < .001, ε^2^ = 0.00	χ^2^ = 560.8, *p* < .001, ε^2^ = 0.02	χ^2^ = 58.1, *p* < .001, ε^2^ = 0.00
**Area of residence**										
Major cities	28,381 (76.1%)	6804 (24.0%)	2049 (7.2%)	1032 (3.6%)	1166 (4.1%)	632 (2.2%)	11,080 (39.0%)	13,343 (47.0%)	20,489 (72.2%)	16,147 (56.9%)
Inner regional	7410 (19.9%)	1049 (14.2%)	402 (5.4%)	175 (2.4%)	189 (2.6%)	103 (1.4%)	3496 (47.2%)	2141 (28.9%)	6183 (83.4%)	4850 (65.5%)
Outer regional	1216 (3.3%)	162 (13.3%)	51 (4.2%)	16 (1.3%)	24 (2.0%)	7 (0.6%)	706 (58.1%)	272 (22.4%)	1020 (83.9%)	804 (66.1%)
Remote	30 (0.1%)	8 (26.7%)	5 (16.7%)	1 (3.3%)	–	–	14 (46.7%)	9 (30.0%)	22 (73.3%)	14 (46.7%)
Very remote	5 (−)	1 (20.0%)	–	1 (20.0%)	–	–	3 (60.0%)	1 (20.0%)	4 (80.0%)	1 (20.0%)
Missing	273	63	14	8	7	3	144	88	201	159
Chi-squared (χ^2^) (Kruskal-Wallis H), p, ε^2a^		χ^2^ = 385.6, *p* < .001, ε^2^ = 0.05	χ^2^ = 48.1, *p* < .001, ε^2^ = 0.02	χ^2^ = 49.8, *p* < .001, ε^2^ = 0.04	χ^2^ = 51.9, *p* < .001, ε^2^ = 0.04	χ^2^ = 34.8, *p* < .001, ε^2^ = 0.05	χ^2^ = 307.4, *p* < .001, ε^2^ = 0.02	χ^2^ = 1002.2, *p* < .001, ε^2^ = 0.06	χ^2^ = 449.5, *p* < .001, ε^2^ = 0.02	χ^2^ = 209.7, *p* < .001, ε^2^ = 0.01
**Common law lodged**										
Yes	1407 (3.8%)	1016 (72.2%)	183 (13.0%)	72 (5.1%)	318 (22.6%)	157 (11.1%)	890 (63.2%)	729 (51.8%)	362 (25.7%)	872 (62.0%)
No	35,908 (96.2%)	7071 (19.7%)	2338 (6.5%)	1161 (3.2%)	1068 (3.0%)	588 (1.6%)	14,553 (40.5%)	15,125 (42.1%)	27,557 (76.7%)	21,103 (58.8%)
z statistic (Mann-Whitney U), p, r^a^		z = −46.9, *p* < .001, r = 0.52	z = −9.5, *p* < .001, r = 0.19	z = −3.9, *p* < .001, r = 0.11	z = −38.2, *p* < .001, r = 1.03	z = −25.0, *p* < .001, 0.92	z = −17.0, *p* < .001, r = 0.14	z = −7.2, *p* < .001, r = 0.06	z = −43.2, *p* < .001, r = 0.26	z = −2.4, *p* < .016, r = 0.02

^a^ r is the effect size and ε^2^ (epsilon squared) is the estimate of effect size

Of the claimants with radiological imaging claim payments, most (87%) had a plain radiograph payment, one-quarter had a CT scan, and one in ten had a MRI (Table 2). Compared with females, males were more likely to receive payment for plain radiographs and CT scans. The youngest aged claimants (18–24 years) were significantly more likely to have plain radiograph claim payments compared with older age groups, whereas, claimants aged 35–64 years were more likely than both younger and older age groups to have MRI payments, and the proportion of claimants with CT scans increased with increasing age. Compared to claimants from major cities and remote regions, claimants from regional areas were more likely to receive payments for plain radiographs and CT scans and less likely to receive payments for MRI and Ultrasound (Table 2). Three quarters (*n* = 16,755) of radiology claimants received payment for only a single type of radiology service with most single radiology payments for plain radiographs (86% of single radiology claimants, *n* = 14,347). Of the one in five (*n* = 4214) of radiology claimants with two different types of radiology payments, most received payments for plain radiographs and CT scans (71%, *n* = 2980). Less than 1 % of radiology claimants (*n* = 156) received payments for all four types of radiology services.

**Table 2:** Number (row percentage) of radiology claimants receiving specific radiology type claims within each descriptive category

	n	Plain radiograph (x-ray)	MRI	CT	Ultrasound
**Total**	21,975	19,183 (87.3%)	2236 (10.2%)	5511 (25.1%)	1427 (6.5%)
**Sex**					
Male	7881	6888 (87.4%)	819 (10.4%)	2158 (27.4%)	478 (6.1%)
Female	14,088	12,291 (87.2%)	1416 (10.0%)	3350 (23.8%)	948 (6.7%)
z statistic (Mann-Whitney U), p, r^a^		z = − 3.1, *p* < .002, r = 0.02	z = − 1.5, *p* < .134, r = 0.03	z = −6.7, *p* < .001, r = 0.09	z = − 1.3, *p* < .182, r = 0.03
**Age**					
18–24	5411	4989 (92.2%)	243 (4.5%)	1020 (18.9%)	159 (2.9%)
25–34	4824	4207 (87.2%)	455 (9.4%)	1132 (23.5%)	288 (6.0%)
35–44	4206	3542 (84.2%)	571 (13.6%)	1153 (27.4%)	308 (7.3%)
45–54	3576	3046 (85.2%)	550 (15.4%)	1015 (28.4%)	306 (8.6%)
55–64	2233	1891 (84.7%)	282 (12.6%)	662 (29.6%)	216 (9.7%)
> 65	1725	1508 (87.4%)	135 (7.8%)	529 (30.7%)	150 (8.7%)
Chi-squared (χ^2^) (Kruskal-Wallis H), p, ε^2a^		χ^2^ = 112.5, *p* < .001, ε^2^ = 0.01	χ^2^ = 363.0, *p* < .001, ε^2^ = 0.16	χ^2^ = 200.8, *p* < .001, ε^2^ = 0.04	χ^2^ = 199.7, *p* < .001, ε^2^ = 0.14
**Area of residence**					
Major cities	16,147	13,938 (86.3%)	1844 (11.4%)	3925 (24.3%)	1138 (7.0%)
Inner regional	4850	4381 (90.3%)	313 (6.5%)	1304 (26.9%)	232 (4.8%)
Outer regional	804	717 (89.2%)	52 (6.5%)	233 (29.0%)	45 (5.6%)
Remote	14	11 (78.6%)	3 (21.4%)	3 (21.4%)	–
Very remote	1	1 (100%)	–	–	–
Chi-squared (χ^2^) (Kruskal-Wallis H), p, ε^2a^		χ^2^ = 268.9, *p* < .001, ε^2^ = 0.01	χ^2^ = 61.7, *p* < .001, ε^2^ = 0.03	χ^2^ = 87.1, *p* < .001, ε^2^ = 0.02	χ^2^ = 13.8, *p* < .007, ε^2^ = 0.01
**Common law lodged**					
Yes	872	569 (65.3%)	447 (51.3%)	400 (45.9%)	196 (24.5%)
No	21,103	18,614 (88.2%)	1789 (8.5%)	5111 (24.2%)	1231 (5.8%)
z statistic (Mann-Whitney U), p, r^a^		z = −6.7, *p* < .001, r = 0.04	z = −41.7, *p* < .001, r = 0.88	z = −14.8, *p* < .001, r = 0.20	z = −20.3, *p* < .001, r = 0.53

^a^ r is the effect size and ε^2^ (epsilon squared) is the estimate of effect size

**Fig. 1:**
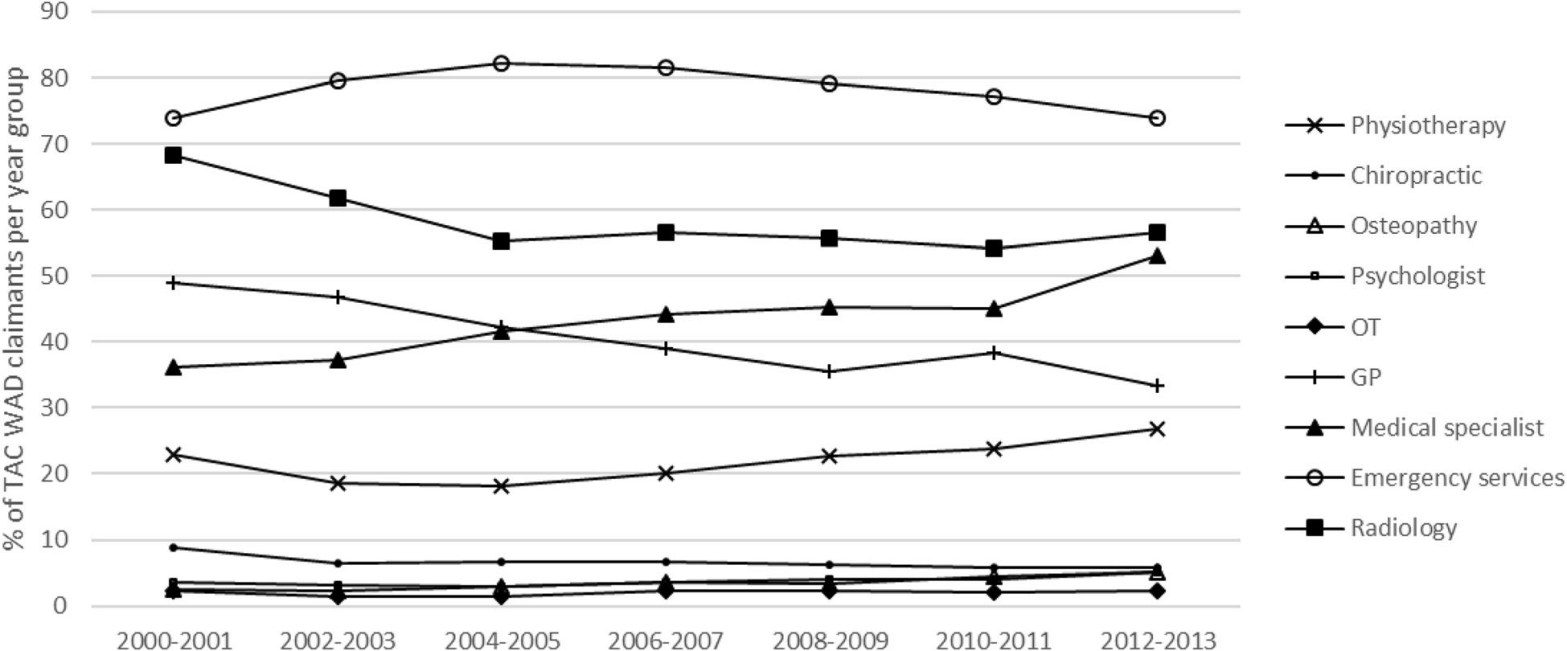
Percentage of TAC WAD claimants with health service claim payments for each accident year group

**Fig. 2:**
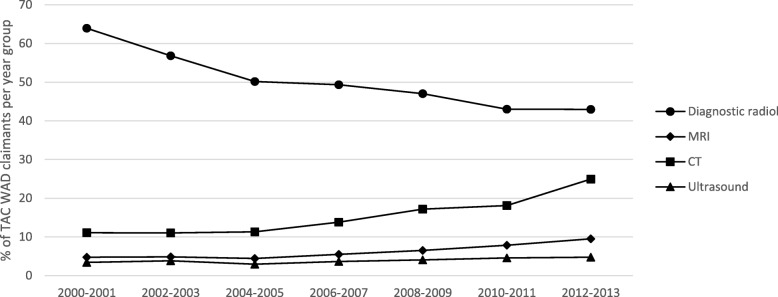
Percentage of TAC WAD claimants with radiology imaging claim payments for each accident year group

The types of health services claim payments changed over the study period. From 2000/2001 to 2012/2013, the percentages of claimants with emergency services and physiotherapy payments remained fairly stable (Fig. 1). Whereas, the percentage of claimants reduced by one-third for chiropractic and GP payments, and by 16% for radiology payments (Fig. 1). Conversely, the percentage of claimants with medical specialist services increased by 47% and increased by 35% (3.7 to 5% of year group claimants) for psychology services (Fig. 1). As shown in Fig. 2, the percentage of claimants with plain radiograph payments decreased by one-third from the beginning to the end of the study period, whereas, the percentage of claimants with MRI payments doubled and the percentage of claimants with CT scan payments increased by 130%.

### Post-injury period

Almost all TAC WAD claimants received a payment for an acute post-injury health service (95%, *n* = 35,348) and approximately one-third (29%, *n* = 10,871) received a payment for a chronic post-injury health service (Table 3). During the acute post-injury period, over four in five acute post-injury claimants had an emergency services payment, over one in two had a radiology payment, and over one in three had a payment for a medical specialist or GP (Table 3). During the chronic post-injury period: over three of five claimants had a physiotherapy payment, almost one-half a GP payment, one in five a chiropractor payment, and just over one in ten had a psychology payment. In addition, over one in three continued to have payments for medical specialist or radiology services. At both post-injury periods, more females than males claimed for all allied health services (except OT at acute post-injury). Whereas, during the chronic recovery period, a greater proportion of males compared to females had medical payments for GP, medical specialist and radiology services. For females and males, the median number of payments per claimant were highest for physiotherapy and chiropractic services during both acute and chronic post-injury periods (Table 3).

**Table 3:** Data are presented for total, acute and chronic claims and include total number of service group claimants, and details relating to the number of service claim payments ([interquartile range]) per claimant within each service group, and for female and male claimants

	Acute Post-injury Period						Chronic Post-injury Period					
	Total		Female		Male		Total		Female		Male	
	Claimants n (column %)	Median claims n [IQR]	Claimants n (column %)	Median claims n [IQR]	Claimants n (column %)	Median claims n [IQR]	Claimants n (column %)	Median claims n [IQR]	Claimants n (column %)	Median claims n [IQR]	Claimants n (column %)	Median claims n [IQR]
Total	35,348	4 [3,6]	22,910	4 [3,6]	12,427	4 [3,5]	10,871	24 [8,55]	7414	26 [8,56]	3453	21 [7,53]
Physiotherapy	5281 (14.9%)	7 [3,11]	3760 (16.4%)	7 [3,11]	1519 (12.2%)	6 [3,11]	7002 (64.4%)	20 [8,43]	4911 (66.2%)	21 [8,44]	2089 (60.5%)	19 [7,41]
Chiropractic	1411 (4.0%)	6 [3,11]	1008 (4.4%)	5 [3,11]	401 (3.2%)	6 [3,11]	2229 (20.5%)	20 [7,42]	1568 (21.1%)	20 [7,41]	660 (19.1%)	21 [8,45]
Osteopathy	594 (1.7%)	4 [2, 7]	467 (2.0%)	4 [2,7]	127 (1.0%)	4 [2,6]	1090 (10.0%)	14 [5,29]	851 (11.5%)	14 [6,29]	238 (6.9%)	12 [4,29]
Psychologist	382 (1.1%)	3 [2,5]	292 (1.3%)	3 [2,5]	90 (0.7%)	2 [1,3]	1301 (12.0%)	10 [4,22]	953 (12.9%)	10 [4,22]	348 (10.1%)	9 [4,21]
OT	105 (0.3%)	2 [2,3]	64 (0.3%)	2 [2,4]	41 (0.3%)	2 [2,3]	698 (6.4%)	5 [2,18]	534 (7.2%)	5 [2,18]	164 (4.7%)	6 [2,18]
GP	13,040 (36.9%)	1 [1,1]	8418 (36.7%)	1 [1,2]	4619 (37.2%)	1 [1,1]	5228 (48.1%)	5 [2,14]	3485 (47.0%)	5 [2,14]	1742 (50.4%)	6 [2,15]
Medical Specialist	13,549 (38.3%)	1 [1,1]	8624 (37.6%)	1 [1,1]	4922 (39.6%)	1 [1,1]	3349 (30.8%)	3 [1,5]	2129 (28.7%)	3 [1,5]	1219 (35.3%)	3 [1,6]
Emergency Services	29,072 (82.2%)	2 [1,2]	17,833 (77.8%)	2 [1,2]	9975 (80.3%)	2 [1,2]	161 (1.5%)	1 [1,1]	109 (1.5%)	1 [1,1]	62 (1.8%)	1 [1,1]
Radiology services - total	19,776 (55.9%)	2 [1,3]	12,648 (55.2%)	2 [1,3]	7122 (57.3%)	2[1,3]	3758 (34.6%)	2 [1,3]	2450 (33.0%)	2[1,3]	1307 (37.9%)	2[1,3]

n: number

**Fig. 3:**
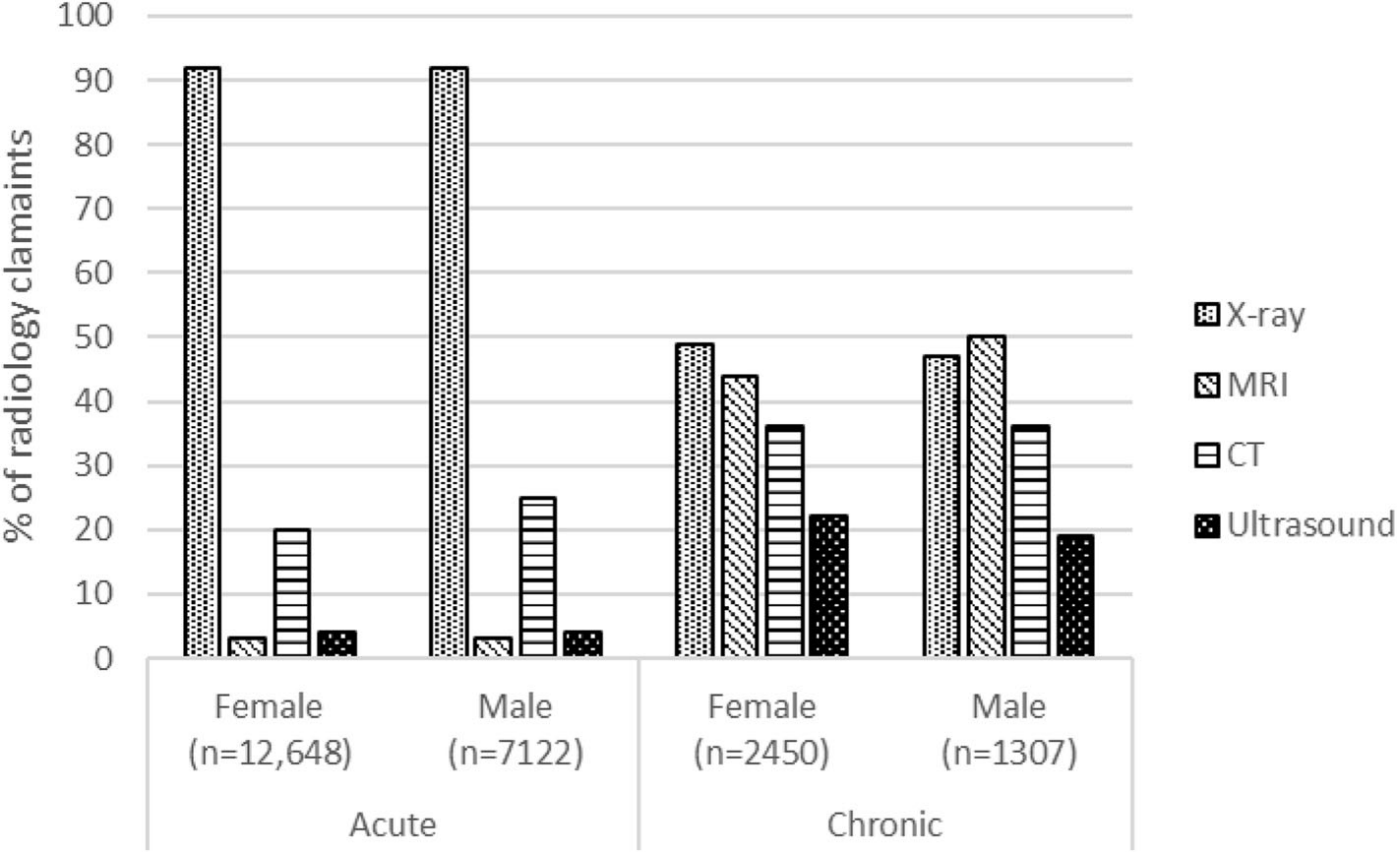
Percentage of acute and chronic female and male radiology claimants receiving specific types of imaging

The types of radiological imaging claimed differed between the acute and chronic post-injury periods. During the acute post-injury period, plain radiographs were claimed by most female and male radiology claimants, although one-quarter of male and one-fifth of female acute radiology claimants had CT scans (Fig. 3). Whereas, MRI, CT scans and ultrasound were claimed much more frequently during the chronic post-injury period: 50% of male and 44% of female chronic radiology claimants had a MRI payment; and over one-third of both male and female claimants had a CT scan. Payments for plain radiographs remained high during the chronic post-injury period (Fig. 3).

### Health service use profiles

It was surmised that claimants with only acute post-injury payments (70.9% of claimants, *n* = 26,444) may have recovered compared with claimants who also had claims during the chronic post-injury period. Logistic regression showed that claimants with only acute payments were more likely to be male (OR = 1.31; 95% CI: 1.24 to 1.38); compared with 18–24 year olds, significantly less likely to be 45–54 years (OR = 0.86; 95% CI:0.78 to 0.94), 55–64 years (OR = 0.79; 95% CI: 0.71 to 0.87) and > 65 years (OR = 0.74; 95% CI: 0.66 to 0.82); compared to the beginning of the study (2000/2001), were significantly more likely to have an accident in the years 2004/2005 (OR = 1.32; 95% CI: 1.20 to 1.45), 2006/2007 (OR = 1.45 95% CI:1.32 to 1.58), 2008/2009 (OR = 1.32; CI:1.20 to 1.45), and 2010/2011 (OR = 1.24; 95% CI: 1.13 to 1.37); and significant less likely to have a common law claim (OR = 0.06; 95% CI: 0.05 to 0.07). Although significant, (× 2 (df = 14) =3443.1, *p* < .000), the model only explained between 8.8% (Cox & Snell R2) and 12.6% (Nagelkerke R2) of the variance.

On the other hand, it was supposed that high service use (e.g. highest quartile, number of health service payments > 10 (*n* = 7779)) may have been associated with greater on-going pain and disability. Logistic regression showed that claimants with highest quartile of health services payments were more likely to be female (OR = 0.74; 95% CI: 0.70 to 0.78); and compared with 18–24 year olds, significantly more likely to be 45–54 years (OR = 1.18; 95% CI: 1.06 to 1.31), 55–64 years (OR = 1.30; 95% CI: 1.17 to 1.45) and > 65 years (OR = 1.41; 95% CI: 1.25 to 1.59); compared to the beginning of the study (2000/2001), were significantly less likely to have an accident in the years 2004/2005 (OR = 0.84; 95% CI:0.76 to 0.93), 2006/2007 (OR = 0.74; CI:0.67 to 0.82), 2008/2009 (OR = 0.80; 95% CI: 0.72 to 0.89), 2010/2011 (OR = 0.81; CI:0.73 to 0.90), and 2012/2013 (OR = 0.87; 95% CI: 0.79 to 0.97); and significantly more likely to have a common law claim (OR = 7.19; 95% CI: 6.41 to 8.06). Although significant (X2 = 2474.36, df = 14, *p* = .000), the model only explained between 6.4% (Cox & Snell R2) and 10.0% (Nagelkerke R2) of the variance.

## Discussion

Individuals with WAD claimed for a range of medical and allied health services. The majority of claimants used emergency services initially. During an acute post-injury period, claimants tended to use medical services with similar numbers of claimants consulting with GPs and medical specialists. Individuals with an on-going need for health services expanded their treatment to include more diverse allied health services while continuing to consult with GPs. Clinical management guidelines for WAD address these types of medical and allied health services through recommendations for treatment, and where indicated, referral to radiological imaging and specific medical and health services [9, 10, 20, 21]. Our current study findings provide a clearer understanding of guideline concordant and discordant health care utilization during both acute and chronic post-injury periods.

Current clinical management guidelines for acute WAD recommend the Canadian C-Spine rule be applied in alert and stable trauma patients [22] to screen for clinically important cervical spine injuries [8, 10]. For individuals found to be at risk of a clinically important cervical spine injury, radiological imaging is oftentimes used to establish a diagnosis. For individuals not at risk of clinically important cervical spine injuries, radiological imaging is not recommended and unnecessary radiological imaging is avoided. Of individuals who present to an ED, clinically important cervical spine injuries occur in approximately 2% of individuals following a blunt trauma (e.g. MVC) [22, 23] and in less than 1% of individuals with an acute whiplash injury [24]. Although it is unknown whether the Canadian C-Spine rule was used in the present study, the apparently high acute post-injury imaging rate of 56% is similar to Canadian [25] and Australian [26] ED imaging rates where successful implementation of the Canadian C-Spine rule resulted in no missed cervical fractures or dislocations.

Most of the acute radiology payments were for x-rays (91%), though one in five claimants had a CT scan and annual CT scan imaging rates doubled from the beginning to end of the study period. Emergent evidence has shown that CT scans are significantly more sensitive than x-rays for identifying cervical spine fractures [27], and are now considered the optimal modality for identifying this type of injury despite the higher radiation levels [28, 29]. The use of x-rays and CT scans during a chronic post-injury phase is unclear since identification of a clinically important cervical spine injury would be evident acutely. Over one-third of chronic post-injury radiology claimants had a CT scan, almost half had an x-ray, and half of male and 44% of females had a MRI. MRI may have been used to screen for neurological complications in individuals who were not recovering. However, the percentage of individuals with neurological complications in this population is usually very small [24, 30, 31]. Importantly, results from imaging during a chronic post-injury phase do not provide additional information that lead to changes in treatment [32]. Given the need to minimise unnecessary costs, exposure to radiation, and possible psychological stress associated with unnecessary radiological imaging, the high use of radiological imaging during a chronic recovery period in the present study is a concern.

The reasons for radiology imaging in the present study are unknown. Perhaps patients with on-going WAD continue to search for a diagnosis to legitimise their pain, and may request or demand additional testing [33, 34]. Clinicians may not be adequately prepared to explain reasons why imaging may be inappropriate and possibly detrimental [35], or may fear missing a fracture [32]. Promoting discussions and education about unnecessary healthcare as advocated by initiatives such as ‘Choosing Wisely ®’ [36, 37] may help both healthcare providers and their patients better understand evidence-based recommendations for tests, treatments and procedures such as radiological imaging.

In Australia, most primary medical care is provided by GPs, the most common medical service provider consulted by individuals with WAD [17, 38]. However, in the present study, slightly more claimants consulted with medical specialists than with GPs during an acute post-injury period. Although reversed during a chronic post-injury period, 35% of male claimants continued to consult with medical specialists. Furthermore, from the beginning to the end of the study period, the percentage of claim payments for medical specialists increased by 50% while GP consultations decreased by one-third. Since 1995, the core clinical guideline recommendations for WAD have remained similar: provide reassurance and advice to encourage return to usual activity and exercise; and avoid immobility collars, [8–10, 20, 21, 39], surgery and pharmaceutical injections [8, 9, 40]. Compared with access to medical specialists, significantly fewer claimants accessed health services likely to encourage an active approach to treatment, such as physiotherapy and chiropractic services [41], during the acute post-injury period. Although the specific type of medical speciality and the reasons for referral to a medical specialist are unknown, and provision of advice to encourage return to usual activity and exercise does not need to come from a physiotherapist or chiropractor, the relatively high referral rates to medical specialists were surprising. Further research is needed to investigate the reasons for referral to medical specialists in this population.

Most previous studies have not differentiated physiotherapy or chiropractor usage rates during acute versus chronic post-injury periods for individuals with WAD, though in another Australian state, 50% received physiotherapy at 9 weeks post-injury [30], and, in Canada, 25% received physiotherapy and 8% received chiropractor services within 6 weeks post- injury [12]. Lower usage rates were found in the present study: only 15% of acute post-injury claimants accessed physiotherapy and 4% accessed chiropractic services. Perhaps, individuals who visited a physiotherapist or chiropractor did not meet the monetary excess required for a TAC service payment and therefore were not included with our data. Alternatively, claimants may have relied on GPs or medical specialist healthcare in the weeks immediately following their injury. Early access to physiotherapy may help to minimise overall healthcare use and promote self-report recovery [12], though overuse of treatments may lead to treatment dependency [12, 14]. Reduced self-report recovery was reported for individuals who had more than six physiotherapy or chiropractor sessions in the first 6 weeks post- whiplash injury [12]. In our study, the volume of consultations appeared to be appropriate during an acute post-injury period (i.e. median = 6 sessions in 12 weeks). The optimal number of treatment sessions during a chronic post-injury period is unknown.

The very high number of physiotherapy and chiropractor sessions per claimant (e.g. 20) during the chronic post-injury period in the present study was higher than previously reported in Australia (e.g. 15) [14, 30]. Consistent with previous research, approximately one-third of WAD claimants appeared to have some level of on-going disability indicated by a health service payment during the chronic post-injury period [2, 6], two-thirds of claimants were female [6, 42, 43], and females were more likely than males to have allied health service payments. Evidence clearly show that females are more likely than males to consult with physiotherapists [14, 44, 45], chiropractors [46], and mental health professionals [47, 48]. Possible gender differences in perceptions of health [49] and reporting of symptoms [45, 49] may result in females seeking more or different help for prevention or illness [49]. Alternatively, males may feel that allied health services, such as physiotherapy or psychology, are less acceptable types of health management [45].

Psychology services were accessed by 1% of claimants during an acute post-injury period, and 13% of females and 10% of males during a chronic post-injury period. To our knowledge, no previous data are available about referral rates of individuals with WAD to psychologists despite evidence that one-quarter of individuals with chronic WAD have a co-morbid mental health disorder/distress [50], acute stress symptoms are prognostic for poor recovery [51], and over 40% of chronic post-injury pharmaceutical claimants from the same data cohort received an anti-depressant [18]. Current clinical WAD management guidelines recommend assessing for post-traumatic stress symptoms where appropriate and onward referral to mental health specialists if required [8]. However, individuals with WAD may be reluctant to consult with psychologists. Individuals with acute WAD questioned the relevance of seeing a psychologist for what they perceived to be a physical injury [52], and individuals with on-going WAD indicated that referral to a psychologist did not align with their treatment expectations [53]. The 40% increase in the percentage of claimants accessing psychology health services over the study period suggests that perceptions of psychology management may be changing in this population. Further research is needed to better understand how and when to appropriately utilize psychological services to improve treatment outcomes, and patient expectations and understanding of psychological health services.

This study provides much needed data about health service use during acute and chronic post-injury periods in individuals with WAD. Nevertheless, limitations exist. Health service use may have been underestimated for two reasons. Firstly, there may have been individuals injured in an MVC who were entitled to claim, but did not lodge a claim for various reasons (e.g., may have had a minor injury that recovered), and hence were not included in the CRD. Secondly, some individuals entitled to make a TAC claim may have received healthcare, but did not meet the monetary excess for reimbursement, and hence were not included in the CRD. However, the monetary excess was likely met for individuals with chronic WAD. Thus, it is likely that the data for the present study captured chronic health service use. Finally, generalisability may be limited since the data were for individuals injured in a single Australian jurisdiction. Given that all individuals with a whiplash injury from an MVC are eligible to claim for health service costs within the no-fault compensation system in Victoria, it is likely that the data represented a general WAD cohort.

## Conclusions

In conclusion, these health service use data provide a better understanding of healthcare service utilization by individuals with WAD during acute and chronic post-injury periods. Apparent concordance with current clinical WAD management guidelines occurred for radiology imaging usage rates during the acute post-injury period, and for physiotherapy and chiropractor services during a chronic post-injury period. Conversely, the low physiotherapy and chiropractic usage during an acute post-injury period, and high referral rates to radiology imaging and medical specialists during the chronic post-injury period appeared discordant with current clinical management guidelines. Strategies are needed to help inform medical health professionals of the current clinical WAD management guidelines to promote early access to health professionals likely to provide an active approach to treatment, and to reduce unnecessary referral to radiology and medical specialists in individuals with on-going WAD.

## Data Availability

Data to support the findings of this study are available from the Institute for Safety, Compensation and Recovery Research (https://www.iscrr.com.au/), however restrictions apply to the availability of these data, which were used under contract for the current study, and so are not publicly available.

## Abbreviations

AU: Australian
CRD: Compensation Research Database
C-Spine: Canadian Cervical Spine rule
CT: Computed tomography
GP: General practitioner
MRI: Magnetic resonance imaging
MVC: Motor vehicle crash
OT: Occupational therapist
QTF: Quebec Task Force
TAC: Transport Accident Commission
WAD: Whiplash associated disorder
